# Sex-Based Disparities in the Transition to Dolutegravir-Based Antiretroviral Therapy in West African HIV Cohorts

**DOI:** 10.1093/ofid/ofae139

**Published:** 2024-03-19

**Authors:** Thierry Tiendrebeogo, Karen Malateste, Armel Poda, Albert Minga, Eugene Messou, Henri Chenal, Oliver Ezechi, Didier K Ekouevi, Igho Ofotokun, Antoine Jaquet, IeDEA West Africa Collaboration, Marcel Djimon Zannou, Armel Poda, Oliver Ezechi, Eugene Messou, Henri Chenal, Kla Albert Minga, Aristophane Tanon, Moussa Seydi, Ephrem Mensah, Caroline Yonaba, Lehila Bagnan Tossa, Jocelyn Dame, Sylvie Marie N’Gbeche, Kouadio Kouakou, Madeleine Amorissani Folquet, François Tanoh Eboua, Fatoumata Dicko Traore, Agatha David, Elom Takassi, Antoine Jaquet, Didier Koumavi Ekouevi, François Dabis, Renaud Becquet, Charlotte Bernard, Karen Malateste, Olivier Marcy, Marie Kerbie Plaisy, Elodie Rabourdin, Thierry Tiendrebeogo, Désiré Dahourou, Sophie Desmonde, Julie Jesson, Valeriane Leroy, Raoul Moh, Jean-Claude Azani, Jean Jacques Koffi, Eric Komena, Maika Bengali, Abdoulaye Cissé, Guy Gnepa, Apollinaire Horo, Simon Boni, Eulalie Kangah, Corinne Moh, Jeanne Eliam, Ighovwerha Ofotokun, Chris Martin, Noelle Benzekri, Geoffrey Goettlieb, Olivia Keiser, Antoine Jaquet, Didier Ekouevi, Ighovwerha Ofotokun, Renaud Becquet, Noelle Benzekri, Charlotte Bernard, Simon Boni, François Dabis, Désiré Dahourou, Sophie Desmonde, Didier Koumavi Ekouevi, Antoine Jaquet, Julie Jesson, Eric Komena, Valeriane Leroy, Karen Malateste, Ighovwerha Ofotokun, Marie Kerbie Plaisy, Elodie Rabourdin, Thierry Tiendrebeogo

**Affiliations:** National Institute for Health and Medical Research UMR 1219, Research Institute for Sustainable Development EMR 271, Bordeaux Population Health Research Centre, University of Bordeaux, Bordeaux, France; National Institute for Health and Medical Research UMR 1219, Research Institute for Sustainable Development EMR 271, Bordeaux Population Health Research Centre, University of Bordeaux, Bordeaux, France; Department of Infectious Diseases, Université Nazi Boni, Bobo-Dioulasso, Burkina Faso; Centre médical de Suivi des Donneurs de Sang, Centre National de Transfusion Sanguine Côte d’Ivoire, Abidjan, Côte d’Ivoire; Centre de Prise en charge de Recherche et de Formation, Abidjan, Côte d’Ivoire; Programme PACCI/ANRS Research Center, Abidjan, Cote d’Ivoire; Département de Dermatologie et d’Infectiologie, Unite de Formation et de Recherche des Sciences Médicales, Université Félix Houphouët Boigny, Abidjan, Côte d’Ivoire; Virology Laboratory, Integrated Centre for Bioclinical Research in Abidjan, Abidjan, Côte d’Ivoire; Office of the Central Secretariat, Nigeria Institute for Medical Research, Lagos, Nigeria; National Institute for Health and Medical Research UMR 1219, Research Institute for Sustainable Development EMR 271, Bordeaux Population Health Research Centre, University of Bordeaux, Bordeaux, France; Département de Santé Publique, Université de Lomé, Lomé, Togo; Division of Infectious Diseases, Department of Medicine, School of Medicine, Emory University, Atlanta, Georgia, USA; National Institute for Health and Medical Research UMR 1219, Research Institute for Sustainable Development EMR 271, Bordeaux Population Health Research Centre, University of Bordeaux, Bordeaux, France

**Keywords:** dolutegravir, HIV, sex disparities, switch, West Africa

## Abstract

Transition to dolutegravir among 21 167 individuals experienced in antiretroviral therapy in West Africa showed heterogeneous timelines and patterns. Initially reported sex disparities tended to catch up over time with persisting disparities, according to contributing HIV clinics. Key factors facilitating dolutegravir switch were male sex, age <50 years, viral suppression, and regimens not based on protease inhibitors.

Dolutegravir (DTG), a second-generation integrase strand transfer inhibitor, has become a first-line antiretroviral therapy (ART) option for HIV treatment due to its high efficacy, tolerability, and resistance profile [1–4]. In 2018, the World Health Organization (WHO) recommended DTG-based ART (DTG-ART) as preferred first-line ART globally [5]. Subsequently, many countries in sub-Saharan Africa, including West Africa, introduced DTG-ART through their national HIV programs. However, initial safety concerns emerged about potential neural tube defects in infants born to women taking DTG at conception [6]. In its July 2018 interim guidance, the WHO advised caution for using DTG in women and adolescent girls of childbearing age. By July 2019, with more safety data available and risk-benefit analyses from modeling studies, the WHO recommended DTG as the preferred drug for first- and second-line ART in all populations, including pregnant women and those of childbearing age [7]. Since then, most countries in West Africa have started transitioning to DTG-ART [8]. While early reports from low- and middle-income countries have shown early gender disparities in access to DTG, there is limited information on the transition's progress and no clear evidence that the gap in access to DTG among women has closed over time [9–12]. We aimed to assess DTG transition and examine predictors of the switch to DTG among ART-experienced persons living with HIV (PLHIV) in West Africa during the 2019–2021 period.

## METHODS

### Data Sources

Deidentified standardized routine clinical data were used as part of the International Epidemiology Databases to Evaluate AIDS (IeDEA) West Africa Collaboration (http://iedea-wa.org). Five HIV clinics in 3 countries with sufficiently updated data contributed to the present analysis: the Centre medical de Suivi des Donneurs de Sang (CMSDS), the Centre de Prise en charge de Recherche et de Formation (CePReF), and the Virology Laboratory, Integrated Centre for Bioclinical Research (CIRBA), Abidjan, Côte d’Ivoire; the Souro Sanou University Hospital Center (CHUSS), Bobo-Dioulasso, Burkina Faso; and the National Institute of Medical Research (NIMR), Lagos, Nigeria. All participating sites are located in urban or periurban areas, and all actively engage in HIV research. The CHUSS and NIMR clinics have academic medical center affiliations. Populations served across these facilities are typically of lower-middle socioeconomic status.

### Study Design and Population

A retrospective cohort analysis was conducted. We included all treatment-experienced PLHIV aged ≥16 years who were receiving care at 1 of the participating HIV clinics. The baseline follow-up visit was defined as the closest routine visit to the date of DTG introduction at each study site. Participants were followed until database closure. The study period ranged from 1 January 2019 to 30 June 2021.

### Outcome and Covariates

The outcome of interest was DTG-ART switch, defined as the switch from a non-DTG regimen to a DTG-ART regimen. Covariates included sex assigned at birth (sex), age categories, duration undergoing ART, CD4 count, virologic status at the date of DTG introduction at the site level (DISL), and contributing HIV clinic. For CD4 count and HIV viral load, the closest measure to the date of DISL within a window of 6 months was retained. Missing data (CD4 count and HIV viral load) were included in a missing value category for descriptive and regression analyses.

### Statistical Analysis

Baseline characteristics of the study population were described in medians and frequencies. Cumulative incidence functions for DTG switch among ART-experienced individuals were computed over the follow-up period, stratified by sex in each cohort. However, we depicted all cumulative incidence curves on a common timeline across sites, with the origin set at 1 January 2019, to highlight the site-specific introduction of DTG along the same calendar timeline. Predictors of the switch to DTG were explored through cause-specific Cox proportional hazards models, with transfer, death, and loss to follow-up as competing risk events and by treating the HIV program as a random effect. To meet the proportional hazards assumption for the variable sex, the overall follow-up time was divided into 2 periods in separate Cox models: the early period from baseline to month 15 (first period) and the late period from month 15 to the database closure date (second period).

### Ethical Approval

The IeDEA West Africa Collaboration obtained authorization from ethics committees in France (IRB00012788), Côte-d’Ivoire (IRB00009111), Burkina Faso (IRB00004738), and Nigeria (IRB00003224). Written informed consent requirements were deferred to the local institutional review boards.

## RESULTS

### Studied Populations

A total of 21 167 ART-experienced PLHIV were included, mainly female (69.4%), with a median age of 45 years (IQR, 39–52) at DISL and a median follow-up of 8 years (IQR, 4–11) since ART initiation. HIV viral load at the time of DISL was documented in 49.5% of PLHIV and was detectable in 11.7% of them. Antiretroviral regimens were based on efavirenz (63.0%), nevirapine (14.9%), and protease inhibitor (21.2%).

### DTG Switch Among ART-Experienced Individuals

**Figure 1.**
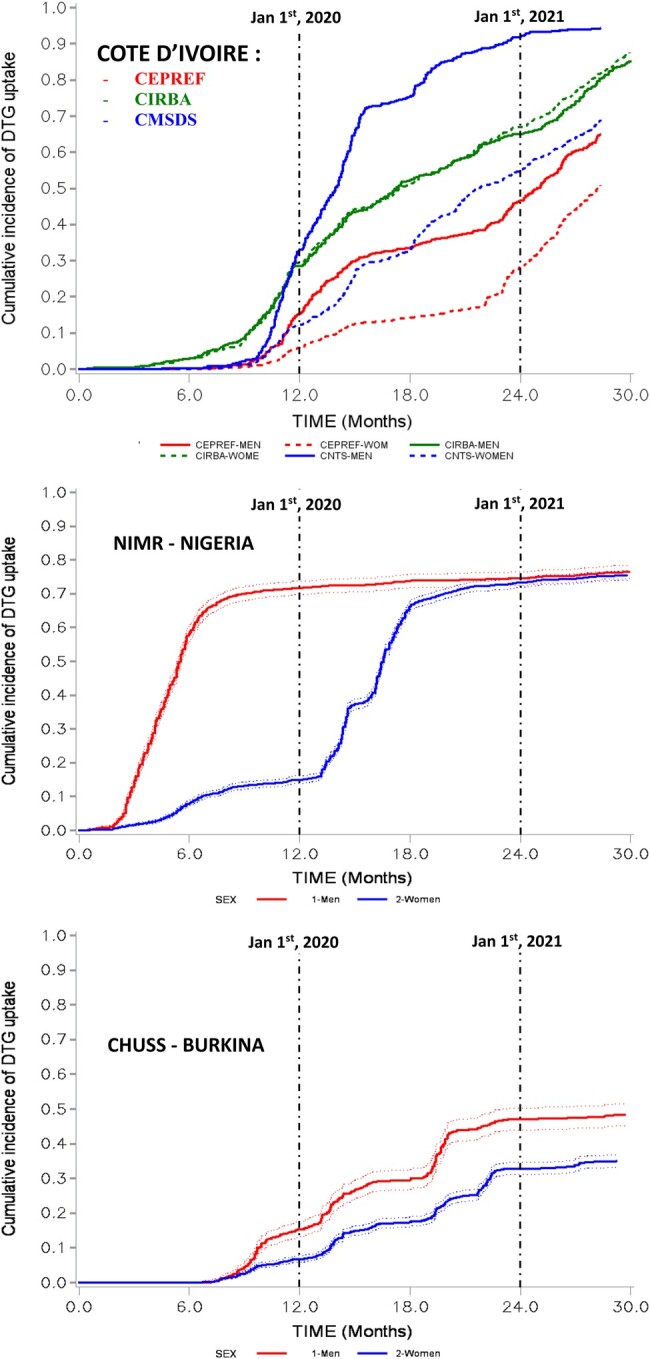
Cumulative incidence function of the switch to dolutegravir (DTG) by sex according to participating cohorts and countries. IeDEA West Africa Collaboration, 2019–2021. CEPREF, Centre de Prise en charge de Recherche et de Formation; CHUSS, Souro Sanou University Hospital Center; CIRBA, Integrated Centre for Bioclinical Research; CMSDS, Centre medical de Suivi des Donneurs de Sang; NIMR, National Institute of Medical Research.

Transition to DTG-ART regimens started in 2019 in all contributing sites. Patterns of DTG rollout differed among and within countries: some cohorts (CePReF, CMSDS, NIMR) showed an initial rapid increase in DTG switch between men and women, followed by a catchup phase among women in the later period; others (CHUSS) showed a persistent gap in women accessing DTG while no sex differences were found in DTG switch in the CIRBA cohort. As of June 2021, DTG switch in men reached 94% at CMSDS, 65% at CePReF, and 48.4% at CHUSS, while for women, corresponding figures were 69%, 51%, and 35%. CIRBA and NIMR exhibited similar rates for sexes, at 85% and 76% of DTG switch, respectively (Figure 1). Stratifying the present analysis on age found that the sex disparity in the DTG-ART switch was observed only in patients aged <50 years.

### Predictors of the Switch to DTG

Cox regression analysis—adjusted for sex, age, ART regimen, and virologic status—showed that male sex was associated with a higher probability of switching to DTG during the first period (adjusted hazard ratio, 5.23; 95% CI, 4.92–5.57) particularly among participants aged <50 years. This association was reversed in the second period (adjusted hazard ratio, 0.8; 95% CI, .7–.9). Additionally, a suppressed HIV viral load was significantly associated with an increased switch to DTG during both periods as well as receipt of nevirapine- and efavirenz-based ART vs protease inhibitor–based ART (Table 1).

**Table 1.** Predictors of the Uptake of DTG-Based ART During the 30 Months Following DTG Introduction at the Site Level: IeDEA West Africa Collaboration, 2019–2021

	Period 1 (Months 0-15)					Period 2 (Months 15-30)				
	PY	IR/100 PY	aHR^a^	95% CI	*P* Value	PY	IR/100 PY	aHR^a^	95% CI	*P* Value
Sex assigned at birth	…	…	…	…	…					<.001
Female						5675	69.1	1 [Ref]		
Male						1770	51.3	0.78	.72–.84	
Age ≤49 y					.029	…	…	…	…	…
Female	12 741	19.3	1 [Ref]							
Male	3008	66.9	5.23	4.92–5.57						
Age >49 y					<.001	…	…	…	…	…
Female	3030	54.3	1 [Ref]							
Male	2470	59.6	1.08	1.01–1.16						
ART regimen					<.001					<.001
2 NRTIs + PI	4836	30.7	1 [Ref]			1966	48.5	1 [Ref]		
2 NRTIs + EFV	13 437	32.2	1.30	1.22–1.38		4815	68.4	1.68	1.55–1.82	
2 NRTIs + NVP	2790	60.8	3.41	3.15–3.69		620	85.4	2.56	2.29–2.87	
Virologic status^b^					<.001					<.001
Detectable	1218	33.1	1 [Ref]			443	48.7	1 [Ref]		
Suppressed	9259	39.7	1.36	1.18–1.57		3169	72.6	1.36	1.18–1.57	
Unavailable	10 771	32.6	1.18	1.06–1.31		3834	60.3	1.21	1.05–1.40	
CD4 count cells/mm^3^					<.001					<.001
<350	1591	41.3	1 [Ref]			575	43.9	1 [Ref]		
350–500	1617	45.9	0.87	.78–.97		557	54.2	0.98	.83–1.15	
>500	5125	42.7	0.99	.91–1.08		1642	72.7	1.25	1.10–1.41	
Unavailable	12 917	30.0	0.84	.78–.91		4671	65.2	1.09	.96–1.23	

Participants included adults undergoing non-DTG ART in West Africa who were HIV positive.

Abbreviations: aHR, adjusted hazard ratio; ART, antiretroviral therapy; DTG, dolutegravir; EFV, efavirenz; IR/100 PY, incidence rate/100 person-years; NVP, nevirapine; NRTI, nucleoside reverse transcriptase inhibitor; PI, protease inhibitor; Ref, reference.

^a^Adjusted cause-specific hazard ratio estimated by Cox regression models.

^b^Virologic status at DTG introduction in the site.

## DISCUSSION

In this study of 21 167 ART-experienced PLHIV, the DTG-ART transition in West Africa revealed marked variations in progress toward the transition according to contributing cohorts. Initial rollout was generally faster in men, although women exhibited catchup phases. In our multivariate analyses, male sex was associated with DTG switch in the early period following its introduction, but the association then reversed in the later period supporting the catchup of women in accessing DTG.

Previous studies reported sex differences in the real-world rollout of DTG [9, 11, 12]. In early years, Romo et al, using data from other regions of the IeDEA consortium, observed persisting sex difference in access to DTG as of March 2020, despite the WHO restrictions being lifted [11]. Completing the picture on the DTG transition in the IeDEA consortium, our study confirms early concerns in access to DTG for women in West Africa but offers reassuring information with regard to DTG switch in women, which has recently increased globally, progressively closing the gap between men and women. But our results have also highlighted heterogeneity in DTG transition among and within countries in real-world contexts. Clinicians were often the main decision makers regarding transitions to DTG. Heterogeneity among clinics may have stemmed primarily from initial clinician reluctance to switch certain groups, in addition to clinic management preferences to end existing drug stocks prior to the switch, rather than any unavailability or gap in the DTG supply chain. Other potential explanatory factors include site-specific staffing patterns, variability in health care infrastructure, and inconsistent diffusion of updated safety guidance. Heterogeneity in access to DTG was also reported by Shah et al in Uganda, Kenya, Tanzania, and Nigeria; they identified a sex-based gap, with disparity between cohorts [12]. In South Africa, Dorward et al recently documented a catchup between women and men but did not highlight any heterogeneity in DTG switch across HIV clinics [9].

Aside sex and age, likely linked to the WHO's safety alert, a suppressed HIV viral load was significantly associated with an increased switch to DTG. This could be attributed to practices among HIV clinics, with some requiring a suppressed HIV viral load before switching to DTG and others not.

The low probability of transitioning to DTG from protease inhibitor–based regimens stemmed from the fact that most patients receiving such regimens were undergoing second- or third-line therapies, whereas guidelines at the time designated DTG as a preferred first-line option, with priority for transition given to those on first-line regimens.

This study has some limitations. First, the HIV clinics involved in these analyses may not be fully representative of all HIV facilities in their countries. Second, given that the study period partially overlapped with the COVID-19 pandemic (2020–2021), the rollout may have been affected by restricted health care provision. Finally, as the study database closure was June 2021, we did not capture recent practices in the rollout of DTG. Despite its limitations, this study provides valuable real-world evidence on the rollout of DTG-ART for first- and second-line regimens across diverse HIV clinics in West Africa, adding value to the existing literature. Especially for HIV care providers, identifying demographic and clinical characteristics associated with lower DTG switch can shape targeted counseling and education efforts to increase parity. From a health policy perspective, observing variability in rollout success across facilities underscores the need for coordinated planning and monitoring to systematically address bottlenecks.

## CONCLUSION

Despite initial sex disparity in DTG transition likely due to perinatal safety concerns, the gap is gradually closing in West Africa, although heterogeneity persists across HIV cohorts. Sensitizing providers through training and updating guidance to emphasize the favorable benefit-risk profile of DTG, including that for pregnant women, could help further close sex disparities. Continual monitoring of DTG implementation is essential, not only to ensure universal and equitable access to ART for PLHIV, but also to comprehend disparities, gauge progress, and assess the role of DTG in reducing HIV drug resistance, particularly in resource-constrained settings.

## Notes

***Potential conflicts of interest***. All authors: No reported conflicts.
